# Beclin 1 regulates recycling endosome and is required for skin development in mice

**DOI:** 10.1038/s42003-018-0279-0

**Published:** 2019-01-25

**Authors:** Saori Noguchi, Shinya Honda, Tatsuya Saitoh, Hiroyuki Matsumura, Emi Nishimura, Shizuo Akira, Shigeomi Shimizu

**Affiliations:** 1 Department of Pathological Cell Biology, Medical Research Institute, Tokyo Medical and Dental University, 1-5-45 Yushima, Bunkyo-ku, Tokyo, 113-8510 Japan; 2 Laboratory of Bioresponse Regulation, Graduate School of Pharmaceutical Sciences, Osaka University, 1-6 Yamada-oka, Suita, Osaka, 565-0871 Japan; 3 Division of Inflammation Biology, Institute for Enzyme Research, Tokushima University, 3-18-15 Kuramoto-cho, Tokushima, 770-8503 Japan; 4 Stem Cell Biology, Medical Research Institute, Tokyo Medical and Dental University, 1-5-45 Yushima, Bunkyo-ku, Tokyo, 113-8510 Japan; 5 Laboratory of Host Defense, Immunology Frontier Research Center, Osaka University, 3-1 Yamada-oka, Suita, Osaka, 565-0871 Japan

## Abstract

Beclin 1 is a key regulator of autophagy and endocytosis. However, its autophagy-independent functions remain poorly understood. Here, we report that Beclin 1 regulates recycling endosome and is required for skin development in vivo. We first established keratinocyte-specific Beclin 1-knockout mice and found that these mutant mice died owing to severe impairment of epidermal barrier. Beclin 1 plays a role in autophagy and the endocytic pathway in cooperation with Atg14 and UVRAG, respectively, and keratinocyte-specific Atg14-knockout mice do not show any abnormal phenotypes, suggesting that Beclin 1 has a role in skin development via the endocytic pathway. Furthermore, we found that Beclin 1 deficiency causes mislocalization of integrins via a defect of recycling endosome, abnormal cell detachment of basal cells and their immature differentiation, and abnormal skin development. These results provide the first genetic evidence showing the roles of Beclin 1 in recycling endosome and skin development.

## Abstract (web summary)

Saori Noguchi et al. demonstrate the role of Beclin 1 for recycling endosome during skin development, using keratinocyte-specific Beclin 1-knockout mice. This study suggests that a key autophagy player Beclin 1 is also important for skin formation by ensuring a proper localization of integrins.

## Introduction

The epidermis of the skin is stratified epithelium and consists of four layers; namely, the basal layer, spinous layer, granular layer, and stratum corneum. At each stage of differentiation, keratinocytes express specific keratins, such as keratin 1 (K1) and keratin 10 (K10) in the spinous and granular layers, and keratin 5 (K5) and keratin 14 (K14) in the basal layer^1^. In epithelial tissue, only cells of the basal layer, which is the nearest layer to the dermis can divide, by which they contribute to the formation of normal epithelial tissue. There are two types of division; i.e., symmetric and asymmetric division. The former type of division contributes to the expansion of the skin area by dividing in an axis parallel to the basal membrane. In contrast, the latter type of division promotes multiple stratification of the epidermis by dividing in an axis perpendicular to the basal membrane^2^. Cells that lose adhesion from the basal membrane by asymmetric division become corneocytes by a terminal differentiation process. Corneocytes are characterized by the loss of all their organelles, as well as by their cornified cell envelope, which is a highly insoluble structure on the inside of the plasma membrane^3^. Loricrin and involucrin are important molecules for keratinization that provide this envelope^4,5^. Filaggrin also has an important role by facilitating the aggregation of keratin intermediate filaments, by which keratin filaments generate a network of two-dimensional sheets that can perform strong barrier functions^6^.

Autophagy is a catabolic process in which cellular contents, including proteins and even entire organelles, are degraded in autophagic vacuoles. Autophagy continuously occurs at low levels and is activated by a variety of cellular events, including cell differentiation^7,8^. The molecular basis of autophagy has been extensively analyzed and several essential genes have been identified, including Ulk1, Beclin 1, and Atg5. Because organellar elimination is associated with keratinocyte differentiation^9,10^, autophagy may contribute to this event. Unexpectedly, however, keratinocyte-specific Atg5-knockout mice, in which autophagy is largely inhibited in epithelial cells, did not show any notable phenotypes^11^.

Beclin 1 is a coiled-coil protein that is a well-known regulator of autophagy in mammalian cells^12,13^. It is a component of the multiprotein complex phosphatidylinositol-3-kinase (PI3K) class III, which generates phosphatidylinositol-3-phosphate (PI3P), an important molecule for membrane trafficking^14^. Beclin 1 is involved in the autophagy machinery together with Atg14 by promoting membrane invagination and by inducing maturation of both autophagosomes and phagosomes. Beclin 1 also has a role in regulating the endocytic pathway together with UVRAG, instead of Atg14^15^. The endocytic pathway involves distinct small vesicles, which internalize molecules from the plasma membrane (early endosomes) and recycle them back (recycling endosomes), or deliver them to lysosomes (late endosomes). Among these endosomes, Beclin 1 has been mainly reported to be involved in the regulation of early endosomes^16,17^, which contain a large amount of PI3P. This is reasonable because Beclin 1 is a component of PI3K and contributes to the generation of PI3P. There are several reports describing the role of Beclin 1 in other types of endosomes^18^; however, genetic and in vivo evidence has been lacking.

To clarify whether or not autophagy and Beclin 1 are involved in skin development, we generated keratinocyte-specific Beclin 1- and Atg14-deficient mice, and analyzed the role of Beclin 1 in skin formation. We demonstrate that Beclin 1 controls the location of integrins through the regulation of recycling endosomes, and is crucial for skin development.

## Results

### Impaired skin barrier function in Beclin 1-deficient mice

**Fig. 1:**
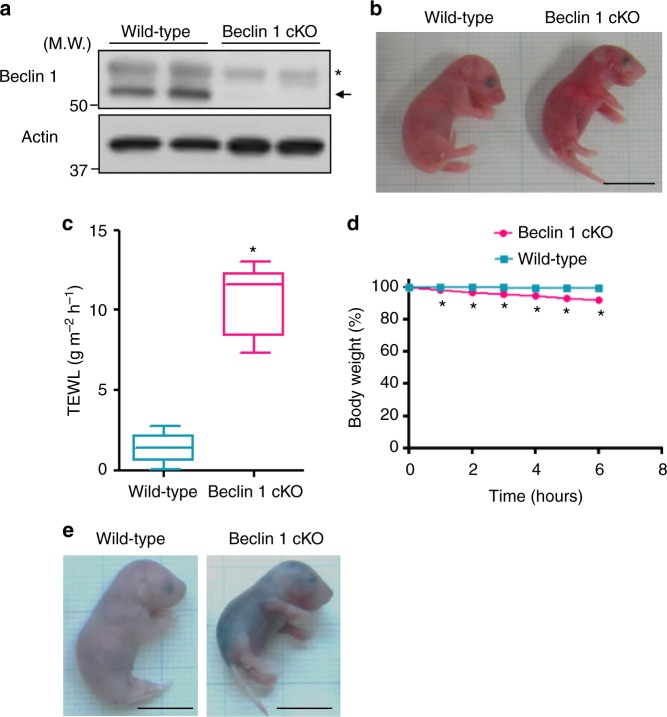
Phenotypes of Beclin 1 cKO mice. **a** Expression of Beclin 1 in the dorsal skin of E18.5 embryos of the indicated mice. Actin was used as a loading control. Arrow indicates the band of Beclin 1. Asterisk indicates a nonspecific band. Representative images of five independent experiments are shown. **b** Gross appearance of Beclin 1 cKO neonatal mice is shown. Scale bars = 1 cm. **c** TEWL assay of neonatal mice. High TEWL values indicate water loss from the epidermis (*n* = 8, mean ± SD). Asterisk indicates a significant difference at *p* < 0.0001. (Student’s *t* test) **d** Body weight loss in Beclin 1 cKO neonatal mice (*n* = 4, mean ± SD). Asterisks indicate a significant difference at *p* < 0.0001. (ANOVA) **e** Loss of skin barrier function in the epidermis of Beclin 1 cKO neonates. Toluidine Blue permeability assay showing the blue staining of a Beclin 1 cKO neonate, which shows impaired barrier function. Scale bars = 1 cm. Uncropped immunoblot images are provided in Supplementary Figure 4a. Data sources for **c** and **d** are provided in Supplementary Data 1

To elucidate the role of Beclin 1 in epidermal skin formation, we generated Beclin 1^flox/flox^ mice, and crossed them with K5-cre transgenic mice to generate keratinocyte-specific Beclin 1-deficient mice (hereafter, referred to as Beclin 1 cKO). Because K5-cre functions in basal cells, Beclin 1 was expected to be completely deleted from epidermal tissue, and which was confirmed by western blotting (Fig. 1a). The mice were born at a Mendelian ratio, but neonatal mice had stiff and shiny skin (Fig. 1b). Furthermore, all neonatal mice died within a day after birth. Measurement of transepidermal water loss (TEWL), which is the amount of water that passively evaporates through the skin to the external environment, indicated extensive water loss in Beclin 1 cKO neonates (Fig. 1c). Consistently, Beclin 1 cKO neonates demonstrated a time-dependent decrease in their body weights (Fig. 1d), which is likely to be owing to their body fluid loss. These results suggested the possible impairment of skin barrier function in Beclin 1 cKO neonates, which was further confirmed by the Toluidine Blue permeability assay. As shown in Fig. 1e, Beclin 1 cKO mice were stained with Toluidine Blue dye due to an increase in skin permeability, indicating that Beclin 1 is crucial for generation of the skin barrier in neonatal mice.

### Beclin 1 cKO mice show aberrant epidermal differentiation

**Fig. 2:**
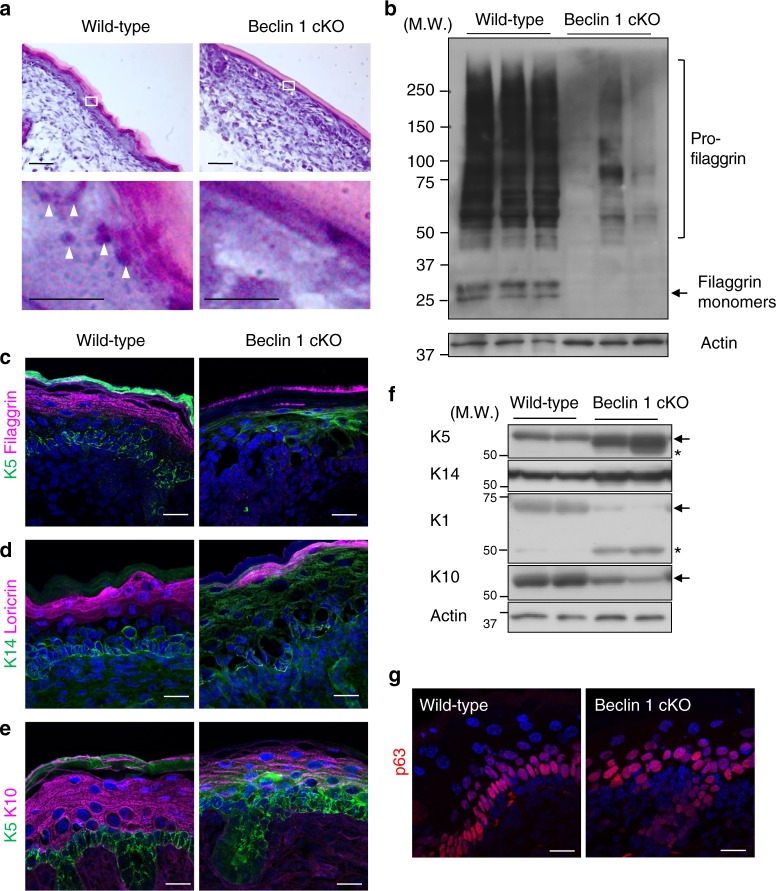
Aberrant epidermal differentiation in Beclin 1 cKO mice. **a** Hematoxylin eosin staining of the dorsal skin of neonatal mice. Scale bars = 50 µm. Regions of the granular layer indicated as white rectangles in the top panels are magnified in the lower panels. Scale bars = 10 µm. Arrowheads indicate keratohyaline granules. **b** Immunoblot analysis of the epidermis of E18.5 mice using an anti-filaggrin antibody. The lower bands indicate filaggrin monomers. The smear signals indicate profilaggrin. Representative images of five independent experiments are shown. **c**–**e** Immunostaining of dorsal skin of E18.5 embryos. Frozen sections were immunostained with anti-K5 (green) and anti-filaggrin (magenta) antibodies in **c**, anti-K14 (green) and anti-loricrin (magenta) antibodies in **d**, and anti-K5 (green) and anti-K10 (magenta) antibodies in **e**. Nuclei were counterstained with DAPI (blue). Scale bars = 20 µm. **f** Immunoblot analysis of the epidermis of E18.5 mice using the indicated antibodies. Arrows indicate the bands of K5, K10, and K1. Asterisks are thought to be cleaved products. Representative images of five independent experiments are shown. **g** Immunostaining of dorsal skin of E18.5 embryos. Frozen sections were immunostained with anti-p63 (red) and counterstained with DAPI to visualize nuclei. Uncropped immunoblot images corresponding to **b** and **f** are provided in Supplementary Figure 4b and c, respectively

To elucidate how Beclin 1 contributes to maintain the skin barrier, we analyzed the histology of skin sections. In the epidermis of Beclin 1 cKO mice, despite the eosin-positive cornified layer looking normal, keratohyaline granules, which are storage sites for profilaggrin, were not generated in the granular layer (Fig. 2a; arrowheads). This was confirmed by the relatively low amount of profilaggrin and filaggrin on Western blot analysis (Fig. 2b) and immunostaining analysis (Fig. 2c; magenta signals) of the epidermis of Beclin 1 cKO mice. Although magenta signals were weakly observed on stratum corneum of Beclin 1 cKO mice, this might be owing to the residual profilaggrin. Because filaggrin is an essential molecule for the keratin filament network, its reduction results in the loss of skin barrier function. Similarly to filaggrin, other proteins in the suprabasal layers, such as loricrin and K10, were substantially reduced in the epidermis of Beclin 1 cKO mice (Fig. 2d, e; magenta signals). In contrast, proteins in the basal layer, such as K5 and K14, which are strictly localized to the basal layer in the normal epidermis, were expressed broadly within the upper layers of the epidermis in Beclin 1 cKO mice (Fig. 2c–e; green signals). Western blot analysis of epidermal tissue confirmed the increased expression of K5 and K14, and decreased expression of K1 and K10 (keratins expressed in spinous and granular layers) in Beclin 1 cKO epidermis (Fig. 2f). We found lower molecular weight bands on sodium dodecyl sulphate-polyacrylamide gel electrophoresis (SDS-PAGE) gels of K5 and K1 from Beclin 1 cKO epidermis, but not from the normal epidermis (Fig. 2f: K5 and K1). This might be owing to previously unknown cleavage. These data indicated that keratinocyte differentiation was largely impaired by the loss of Beclin 1 from basal cells to spindle cells. We next analyzed whether expression levels and localization of p63, a master transcriptional regulator of epithelial development, were altered in Beclin 1 cKO mice. However, immunostaining analysis did not show any abnormalities in p63 expression levels and localization in Beclin 1 cKO mice (Fig. 2g).

We also analyzed the elimination of organelles, which is another marker of keratinocyte differentiation. As shown in Supplementary Figure 1, when we analyzed the localization of mitochondria and Golgi apparatus by the immunostaining of Tom20 and GM130, respectively, we observed normal elimination of organelles in the suprabasal layers of Beclin 1 cKO epidermis. Therefore, elimination of organelles during epidermis development is not affected by Beclin 1 deficiency.

**Fig. 3:**
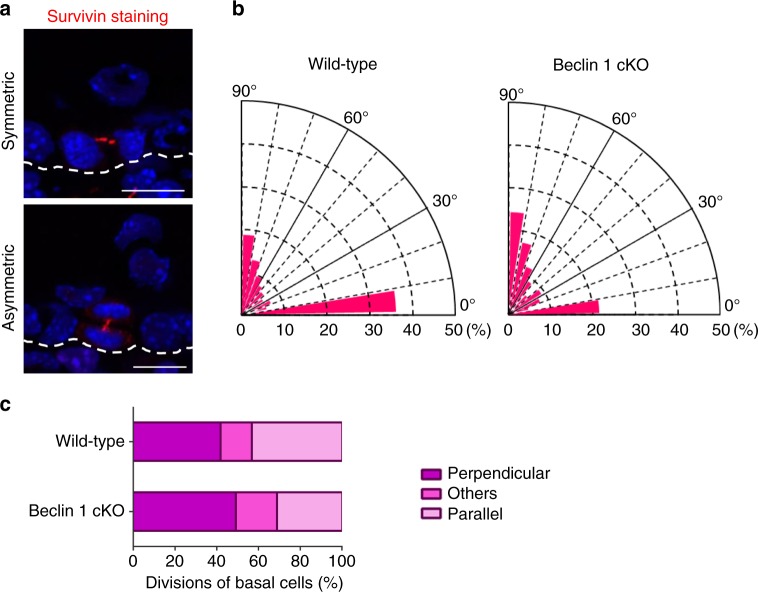
Unbalanced spindle orientation in basal keratinocytes of Beclin 1 cKO mice. **a** Representative images of symmetric (top) and asymmetric (bottom) division in the dorsal skin of E17.5 embryos of wild-type mice. Dividing basal cells were immunostained with an anti-survivin antibody (red). White dotted lines indicate the basal membrane. Scale bars = 10 µm. **b** Angles of spindle orientation relative to the basal membrane were analyzed in multiple fields and shown graphically (*n* = 8). **c** Percentage of basal cell division in the epidermis of Beclin 1 cKO and control mice. The direction of division was classified into three types (parallel: 0–30°, perpendicular: 60–90°, and others: 30–60°) according to the angle from the basal membrane. Data sources for **b** and **c** are provided in Supplementary Data 2

During epithelial tissue formation, cellular differentiation begins with tightly regulated asymmetric division^2^. However, in the epidermis of Beclin 1 cKO mice, basal cell markers and suprabasal cell markers were more broadly expressed and expressed at lower levels in the suprabasal layer, respectively. This indicates a failure of coordination between differentiation and asymmetric division, and hence we analyzed the dimensions of basal cell division by immunofluorescence analysis of survivin, which localizes to midzone microtubules at anaphase/telophase and is thereby widely used to analyze cell division^19^. As shown, basal cells in the normal epidermis were divided equally in the parallel and perpendicular directions (Fig. 3a–c). This balance is crucial for appropriate formation of the epidermis. In contrast, in the epidermis of Beclin 1 cKO mice, the rate of perpendicularly orientated cells was increased (Fig. 3b, c), suggesting that basal cells are prone to move into the suprabasal layer. Earlier asymmetric division is expected to promote immature differentiation, followed by a decrease in keratohyaline granules and the keratin filament network, resulting in the loss of skin barrier functions.

**Fig. 4:**
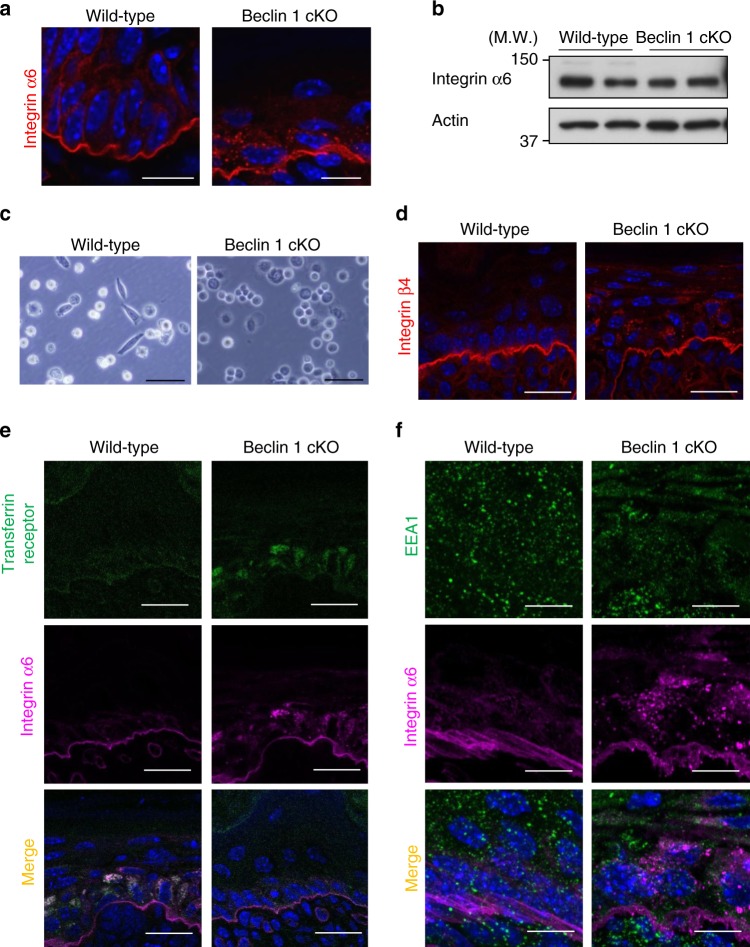
Mislocalization of integrin α6 in the epidermis of Beclin 1 cKO mice. **a** Representative images of integrin α6 mislocalization in the epidermis of Beclin 1 cKO mice. Frozen sections were immunostained with an anti-integrin α6 antibody (red). Nuclei are counterstained with DAPI (blue). Scale bars = 20 µm. **b** Dorsal skin of the indicated E18.5 embryos were lysed and analyzed using an anti-integrin α6 antibody. Actin was used as a loading control. Representative images of two independent experiments are shown. **c** Basal cells were isolated from the epidermis of indicated mice (Postnatal day 0 neonates), scattered into collagen-coated plastic dishes. After 3 h, cells were observed using a phase-contrast microscope. Scale bars = 500 µm. **d** Similar experiments to a were performed using an anti-integrin β4 antibody, instead of an anti-integrin α6 antibody. **e**, **f** Frozen sections of the epidermis of the indicated mice were immunostained with anti-transferrin receptor and anti-integrin α6 antibodies in **e** and with anti-EEA1 and anti-integrin α6 antibodies in **f**. Nuclei were counterstained with DAPI (blue). Scale bars = 10 µm. Uncropped immunoblot images are provided in Supplementary Figure 4d

**Fig. 5:**
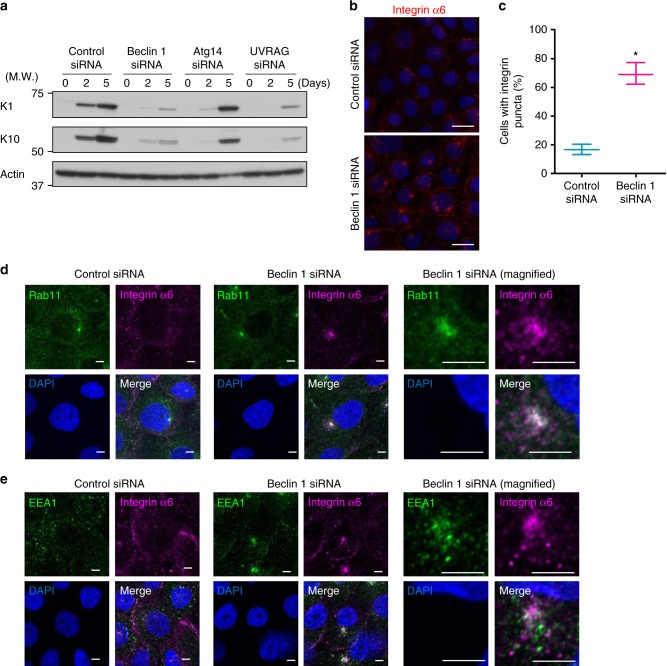
Mislocalization of integrin α6 in Beclin 1-silenced primary keratinocytes. **a** Primary keratinocytes were transfected with the indicated siRNAs, and after 3 days, cells were differentiated by the addition of 2 mM CaCl_2_. Then, cells were harvested and lysed on the indicated days. Immunoblot analysis was performed using the indicated antibodies. Representative images of three independent experiments are shown. **b**, **c** Primary keratinocytes were transfected with the indicated siRNAs, and after 3 days, cells were differentiated by the addition of CaCl_2_. Cells were then assayed for internalized anti-integrin α6 antibodies after 6 h from CaCl_2_ addition. Nuclei were counterstained with DAPI (blue). Representative images are shown in **b**. Scale bars = 20 µm. Note that the puncta of integrin α6 were observed in Beclin 1-silenced cells, but were scarce in control cells. **c** The number of cells with integrin α6 puncta was counted (*n* = 3, mean ± SD). Asterisk indicates a significant difference at *p* < 0.05. (Student’s *t* test) **d** Similar experiments with **b** were performed using anti-Rab11 (green) and anti-integrin α6 (magenta) antibodies. Cells were counterstained with DAPI (blue). Representative images are shown. Scale bars = 5 µm. A magnified image of an integrin α6 punctum in Beclin 1-silenced keratinocytes are shown in the right panels. Scale bars = 5 µm. **e** Similar experiments with **d** were performed using an anti-EEA1 antibody instead of an anti-Rab11 antibody. Uncropped immunoblot images are provided in Supplementary Figure 5a. Data sources for **c** are provided in Supplementary Data 3

Because the loss of Beclin 1 facilitated the early detachment of basal cells from the basement membrane, we hypothesized that there may be a dysfunction of one or more adhesion molecules. Accordingly, we immunostained various adhesion molecules, and found the mislocalization of integrin α6. As shown in Fig. 4a, a considerable amount of integrin α6 accumulated in Beclin 1 cKO basal cells with puncta formation. Because total expression levels of integrin α6 was equivalent between wild-type and Beclin 1 cKO cells (Fig. 4b), the amount of cell surface integrins was expected to be decreased. In fact, when we isolated basal cells from the epidermis of these mice and scattered them onto a plastic dish, the attachment of Beclin 1 cKO epithelial cells was far less than normal epithelial cells (Fig. 4c). Similarly to integrin α6, integrin β4 accumulated in Beclin 1 cKO basal cells showing puncta formation (Fig. 4d). Because integrin α6 and β4 formed puncta within cells, we considered the possible involvement of endosome failure. Integrins are first taken up by early endosomes. Cargo proteins subsequently enter late endosomes to be degraded within lysosomes or recycle back to the plasma membrane via directly or using recycling endosomes. Therefore, we immunostained integrin α6 with marker proteins of each type of endosome. Integrin α6 colocalized with the transferrin receptor, which is a marker of recycling endosomes, in the epidermis of Beclin 1 cKO mice (Fig. 5e). In contrast, it did not colocalize with EEA1 (Fig. 5f), a marker of early endosomes, suggesting that integrins were accumulated in recycling endosomes. Note that EEA1 signals were reduced in Beclin 1 cKO basal cells, and hence early endosomes are also affected by the loss of Beclin 1, as reported previously^16^. These findings indicated that Beclin 1 deficiency causes a disruption of endosomal function, particularly of recycling endosomes, and hence integrin α6 accumulates in recycling endosomes.

### Role of Beclin 1 in a keratinocyte culture model

Keratinocyte differentiation can be reproduced by the addition of Ca^2+^ into cultured keratinocytes^20,21^. The exposure of cultured basal cells to low-Ca^2+^ conditions, followed by replacement to medium containing Ca^2+^ (2 mM) induces keratinocyte differentiation. In fact, we observed the expression of K1 and K10 in control human primary keratinocytes, indicating the successful differentiation of basal cells to spinous or granular cells (Fig. 5a). We performed Beclin 1 silencing in this model. Efficient silencing of Beclin 1 was confirmed by western blotting (Supplementary Figure 2). Similar to the mouse epidermis (Fig. 2f), the expression of K1 and K10 were largely suppressed in Beclin 1-silenced keratinocytes (Fig. 5a). In these cells, we observed puncta formation of integrin α6 in Beclin 1-silenced, but not control, keratinocytes (Fig. 5b, c), confirming the failure of endosomal trafficking by Beclin 1 silencing. Recycling endosomes are known to localize close to the microtubule organizing center, to which Rab11, another recycling endosome marker, was localized (Supplementary Figure 3). Despite this Rab11 localization was equivalently observed irrespective of the presence of Beclin 1 (Supplementary Figure 3), integrin α6 signals were completely merged with Rab11 signals only in Beclin 1-silenced cells (Fig. 5d; magnified image), indicating that integrin α6 trafficking was blocked at the recycling endosomes, consistent with the in vivo data. We also analyzed early endosomes using EEA1. EEA1 signals were observed throughout the cytosol in control cells, whereas they were centrally located in Beclin 1-silenced cells (Fig. 5e), where recycling endosomes localize. Interestingly, however, early endosome signals were very close to but little merged with integrin α6 signals (Fig. 5e; magnified image). These results indicated that early endosome trafficking was also disrupted by the lack of Beclin 1 but successfully transferred cargos to recycling endosomes, and cargos are stored in the recycling endosomes without recycling back.

**Fig. 6:**
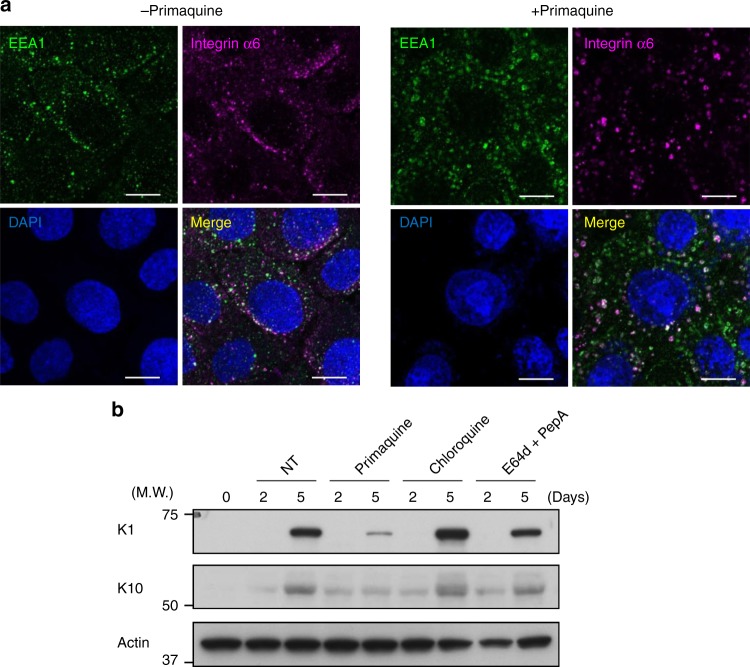
Treatment with vesicular trafficking inhibitor promotes integrin α6 puncta formation and disrupts differentiation in primary keratinocyte. **a** Primary keratinocytes were differentiated by the addition of CaCl_2_ together with primaquine (500 µM). After 6 h, cells were assayed for internalized anti-integrin α6 antibody and  immunostained with anti-EEA1 antibody. Nuclei were counterstained with DAPI (blue). Representative images are shown. Scale bars = 10 µm. **b** Primary keratinocytes were differentiated by the addition of CaCl_2_ together with primaquine (20 µM), Chloroquine (2.5 µM), and E64d (10 µM)/pepstatin (10 µM). On the indicated days, cells were lysed and analyzed for the expression of the indicated proteins by western blot analysis. Representative images of two independent experiments are shown. Uncropped immunoblot images are provided in Supplementary Figure 5b

To elucidate whether a block of endocytic pathway is sufficient to induce the mislocalization of integrins and the failure of keratinocyte differentiation, we added primaquine, an inhibitor of vesicular transport^22^, into cultured keratinocytes. As expected, we observed the mislocalization of integrins (Fig. 6a) and the failure of keratinocyte differentiation as assessed by the expression of K1 and K10 in primaquine-treated cells (Fig. 6b), similar to Beclin 1-silenced cells. Note that integrin α6 signals were completely merged with EEA1 signals (Fig. 6a), which is different from Beclin 1 silencing. This difference might be owing to the strong action of primaquine on early endosomes. These data indicated that endocytic pathway is essential for skin development, in which Beclin 1 has a role in the regulation of recycling endosomes.

**Fig. 7:**
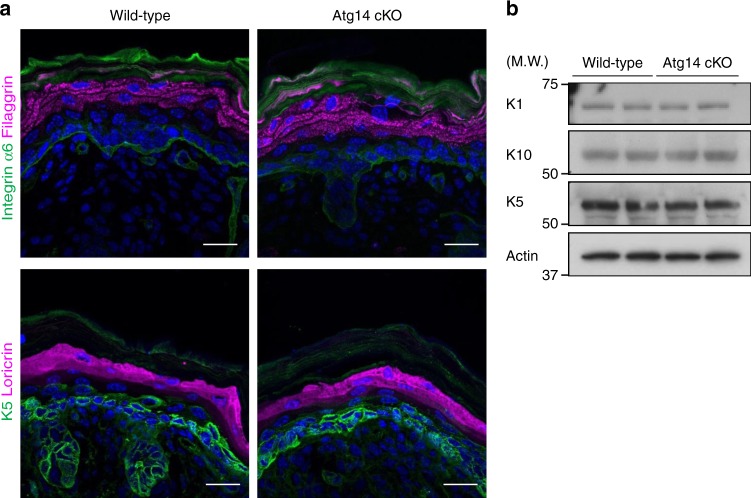
Normal epidermal differentiation in Atg14 cKO mice. **a** Immunostaining of the dorsal skin of neonatal mice. Frozen sections were immunostained with anti-integrin α6 (green) and anti-filaggrin (magenta) antibodies or anti-K5 (green) and anti-loricrin (magenta) antibodies. Nuclei were counterstained with DAPI (blue). Scale bars = 20 µm. **b** Immunoblot analysis of the epidermis of E18.5 mice using the indicated antibodies. Representative images of two independent experiments are shown. Uncropped immunoblot images are provided in Supplementary Figure 5c

We finally addressed whether the failure of keratinocytes in the epidermis of Beclin 1 cKO mice was partly mediated by autophagy. Because Beclin 1 functions in autophagy together with Atg14, we crossed Atg14^flox/flox^ mice with K5-cre mice and generated keratinocyte-specific Atg14-deficient mice (hereafter, referred to as Atg14 cKO). Unlike Beclin 1 cKO mice, Atg14 cKO mice did not show any abnormal phenotypes (Fig. 7a, b), which is consistent with keratinocyte-specific Atg5 KO mice demonstrating only few phenotypes. Furthermore, in a keratinocyte differentiation model, Atg14 silencing did not show any effects, whereas silencing of UVRAG, a molecule functioning in membrane trafficking together with Beclin 1, suppressed differentiation to as similar level to Beclin 1 silencing (Fig. 5a). Furthermore, autophagy inhibitors, such as chloroquine and E64d plus pepstatin A, did not show any effects on keratinocyte differentiation (Fig. 6b). Taken together, our results demonstrate that Beclin 1 is required for skin development through the regulation of endocytic pathway, but not autophagy.

## Discussion

We here demonstrate that Beclin 1 is essential for the development of the epidermis via controlling the proper division of basal cells and integrin trafficking. Beclin 1 is associated with various cellular functions through the regulation of autophagy and membrane trafficking. Beclin 1 functions in autophagy as a component of the Atg14-Beclin 1-Vps34 complex and in membrane trafficking as a component of the UVRAG-Beclin 1-Vps34 complex^15^. Many abnormal phenotypes observed in Beclin 1-deficient mice, including embryonic lethality and tumorigenesis, have been explained from the aspect of autophagy^23,24^. However, the defect of epidermal development is not mediated by defects of autophagy, because keratinocyte-specific Atg14-deficient mice showed no abnormal phenotypes, and a previous study demonstrated no remarkable abnormalities in keratinocyte-specific Atg5 (or Atg7)-deficient mice^11,25^.

In contrast, disturbance of the endocytic pathway is the cause of abnormal epidermal development in Beclin 1 cKO mice. The role of Beclin 1 in the endocytic pathway has been shown in various events. Most of these reports have been on the regulation of early endosomes, i.e., Beclin 1 has a role in neuron viability by regulating endocytosis^17^. These studies show that Beclin 1 is utilized for Rab5 GTPase-associated endosome formation and contributes to EEA1/early endosome localization and late endosome formation. An equivalent function of Beclin 1 has been reported in EGF receptor internalization and degradation, by regulating early and late endosomes in HeLa cells^18^. On the other hand, although there are a few reports that show the involvement of Beclin 1 in the recycling of membrane proteins, such as the phagocytic receptors, CD36 and Trem2 in microglia^26^, and the type I receptor ALK5 in neuronal cells^27^, Beclin 1 function in recycling endosomes remains poorly understood. We here show that the loss of Beclin 1 induced puncta formation of integrins within cells in association with Rab11-positive recycling endosomes. Our data hence provide the first genetic and in vivo evidence showing the crucial role of Beclin 1 in the regulation of recycling endosomes.

Consistently, the failure of recycling endosomes is known to cause defects in skin development. For example, cerebral dysgenesis, neuropathy, ichthyosis, and keratoderma (CEDNIK) syndrome is a rare disease that shows severe developmental failure of the nervous system and the epidermis, which is caused by the decreased expression of SNAP29^28^, a member of the SNARE family that is required for endosome recycling^29^. Interestingly, SNAP29-deficient mice showed similar phenotypes to Beclin 1 cKO mice, i.e., skin appearance, loss of barrier function, and a defect of basal cell differentiation^30^, and hence Beclin 1 may function on recycling endosomes together with SNAP29.

In the epidermis of Beclin 1 cKO mice, dysfunctional integrin recycling appears to be a major cause of skin developmental defects, because integrins have been shown to be essential molecules for skin formation. For example, integrin α6 and β4 knockout mice were shown to die by serious hydroa soon after birth^31,32^. In addition, keratinocyte-specific integrin β1-deficient mice were found to show abnormalities in the division axis of basal cells^2^, similarly our Beclin 1 cKO mice. Thus, integrins have an essential role in normal epidermis development, and the disruption of normal integrin function results in the failure of epidermis generation. The failure of normal skin development in Beclin 1 cKO mice is thought to be mainly mediated by the disruption of endosome recycling, including integrin trafficking, but the skin phenotype of these mice is milder than that of integrin α6 knockout mice. This might be because integrins are expressed in Beclin 1 cKO mice, even though there are problems with integrin trafficking. Taken together, we here showed the essential role of Beclin 1 in skin development via the regulation of recycling endosome.

## Methods

### Animals

Beclin 1-flox mice (C57BL/6N-*Becn1*^*tm1a(KOMP)Wtsi*^) were purchased from the KOMP repository. Atg14^flox/flox^ mice^33^ and K5-cre mice^34^ were kindly provided by Professors S. Akira (Osaka University) and J. Takeda (Osaka University), respectively. All mice were maintained in a specific pathogen-free animal facility at the Laboratory for Recombinant Animals (Medical Research Institute, Tokyo Medical and Dental University, Tokyo, Japan). All experiments were reviewed and approved by the Institutional Animal Care and Use Committee of Tokyo Medical and Dental University, and were conducted according to the committees’ guidelines.

### Chemicals

The following antibodies were used for Western blot and immunofluorescense assays: rabbit anti-Beclin 1 (PD017; Medical & Biological Laboratories ;1:3000) guinea pig anti-K5 (GP-CK5; Progen Biotechnik; 1:5000 for western blot, 1:100 for immunofluorescence), mouse anti-K10 (MAB3230; Millipore; WB: 1:5000, IF: 1:200), mouse anti-K14 (ab7800; Abcam; WB: 1:20,0000, IF: 1:500), rabbit anti-K1 (905204; BioLegend; 1:5000), rabbit anti-loricrin (ab24722; Abcam; 1:2000), rabbit anti-filaggrin (905804; BioLegend; WB: 1:10,000, IF: 1:200), rabbit anti-survivin (#2808; Cell Signaling Technology; 1:100), rat anti-integrin α6 (555734; BD Pharmingen; 1:200), rabbit anti-integrin α6 (#3750; Cell Signaling Technology; 1:1000), anti-integrin β4 (553745; BD Pharmingen; 1:100), rabbit anti-Tom20 (FL-145; Santa Cruz Biotechnology; 1:500), mouse anti-GM130 (610823; BD Pharmingen; 1:100), rat anti-transferrin receptor (553266; BD Pharmingen; 1:100), rabbit anti-EEA1 (#3285 S, Cell Signaling Technology; 1:100), rabbit anti-Rab11 (#5589 S; Cell Signaling Technology; 1:100), mouse anti-γ-tubulin (T6557; Sigma-Aldrich; 1:100), rabbit anti-p63 (H-137; Santa Cruz Biotechnology; 1:50), and mouse anti-actin (#MAB-1501; Millipore; 1:10,000). All other chemicals were purchased from Nacalai Tesque.

### siRNAs

The siRNA sequences used are as follows:

human Beclin 1: CCAAUAAGAUGGGUCUGAAAUUUCA,

human Atg14: CCACUGCAUACCCUCAGGAAUCUAA,

human UVRAG: GACCGAGAGAAAGAUAACAUCUCUA,

Stealth RNAi Negative Control Low GC Duplexes were also used.

### Skin barrier function assays

The Toluidine Blue permeability assay was performed as previously described^35^. In brief, neonatal mice were dehydrated by incubations (1 min each) in 25, 50, and 75% methanol/phosphate-buffered saline (PBS) followed by 1 min in 100% methanol. The embryos were then rehydrated with the same series of methanol solutions (1 min incubations), washed in PBS, and stained for 1 min in 0.0125% toluidine blue O (Chroma Technology, Bellows Falls, VT)/PBS. TEWL of neonatal mice were measured using Vapometer (Delfin Technologies). Water loss was also determined by weight loss in percent from initial birth weight.

### Histology and immunofluorescence

Dorsal skin tissue from mouse embryos or neonatal mice were fixed as described previously^36^. In brief, the epidermis of mice were immersed in ice-cold 4% paraformaldehyde (PFA) in PBS and irradiated three times with a 500 W microwave, and then kept on ice for 20 min. Frozen sections were incubated with primary antibodies and visualized with Alexa 488-conjugated or Alexa555-conjugated secondary antibodies (Life Technologies). Nuclei were counterstained with DAPI. The direction of cell division was analyzed using survivin staining, as described previously^19^. In brief, two daughter cells derived from a single cell were determined using survivin staining. The angle of division was determined by measuring the angle defined by the plane transecting two daughter nuclei relative to the plane of the basal membrane. For immunostaining of γ-tubulin, cells were fixed with 4% PFA in PBS at 4 °C for 20 min followed by 100% methanol treatment at −30 °C for 5 min. All immunofluorescence images were obtained by confocal microscopy (LSM710 Zeiss).

### Western blot analysis

The epidermis of embryos at embryonic day 18.5 (E18.5) were obtained by heat separation, as described previously^37^. In brief, dorsal skin was incubated in PBS at 65 °C for 10 min and placed on ice for 1 min. The epidermis was isolated from the dermis using tweezers. Cultured cells were harvested using scrapers. Epidermis samples or cell samples were lysed in SDS buffer (50 mM Tris-HCl [pH 6.8], 2% SDS) containing a protease inhibitor cocktail (Nacalai Tesque) and subjected to sonication. Total lysates were separated on SDS-PAGE and proteins were transferred from polyacrylamide gels to polyvinylidene difluoride membranes (Millipore). Membranes were blocked with 3% skim milk in tris-bufferd saline (TBS) containing 0.1% Tween-20 (TBS-T) and incubated with primary antibodies. After washes in TBS-T, membranes were incubated with horseradish peroxidase-labeled secondary antibodies and visualized with ChemiLumi One Super reagent (Nacalai Tesque).

### Cell spreading assay

For the isolation of primary mouse keratinocytes, skin was removed from neonatal mice. The skin was floated on 1000 PU/mL dispase (Wako) dermis-side down, and kept at 4 °C overnight. The epidermis was peeled off from the dermis using a tweezers and floated on accutase basal-side down for 7 min at room temperature. The epidermis sheet was rubbed with a tweezers to separate the cells and filter them through a 100 µm cell strainer. Cell suspensions were centrifuged and resuspended in CnT-Prime Medium (CellnTec). The number of live cells was counted by trypan blue staining and 3 × 10^5^ cells were seeded in each well of collagen-coated 12-well plates. Primary keratinocytes were incubated in 37 °C for 3 h and observed by microscopy.

### Cell culture and induction of differentiation

Normal human epidermal keratinocytes (NHEKs) were purchased from Kurabo and were cultured in EpiLife medium (Life Technologies) Supplemented with the HKGS kit (Life Technologies). NHEKs were differentiated by the addition of 2 mM CaCl_2_ to the culture medium when NHEKs reached confluency.

For siRNA transfection, cells were transfected with siRNA to a final concentration of 30 nM using Lipofectamine RNAiMax reagent (Life Technologies) according to the manufacturer’s protocol.

### Analysis of internalized integrin

The integrin internalization assay was performed as previously described^38^. In brief, NHEKs were seeded on collagen-coated glass coverslips in 24-well plates at a density of 2.5 × 10^5^ cells per well. Cells were cultured in EpiLife Medium for 2 days. CaCl_2_ (2 mM) was added to the culture medium and incubated for 6 h at 37 °C. Cells were then incubated for 1 h at 4 °C in EpiLife Medium with 2 mM CaCl_2_ and 10 µg/mL of the anti-integrin α6 antibody (BD Pharmingen) to label the surface integrin α6. Cells were washed twice with EpiLife Medium with 2 mM CaCl_2_ to remove excess antibody and subsequently incubated for 30 min at 37 °C to enable for endocytosis of antibody-labeled integrin α6. Cells were then washed twice with 0.5 M NaCl/0.5% acetic acid to remove the antibodies remaining on the cell surface, and washed with PBS. Cells were then fixed with 4% PFA for 20 min at 4 °C. Cells were permeabilized with 0.05% Triton X-100 in PBS with 1% BSA and visualized with Alexa555-conjugated secondary antibodies.

### Statistical analysis

Results are expressed as the mean ± standard deviation. Statistical analysis was performed using Prism (GraphPad) software. Comparisons of two data sets were performed using the unpaired two-tailed Student’s *t*-tests. All other comparisons of multiple data sets were performed using two-way analysis of variance followed by the Tukey post hoc test. A *p*-value of < 0.05 was considered to indicate a statistically significant difference between two groups.

## Supplementary information

Description of Additional Supplementary Files

Supplementary Information

Supplementary Data 1

Supplementary Data 2

Supplementary Data 3

## Data Availability

All data generated or analyzed during this study are included in this published article and its supplementary information files. The source data underlying the graphs presented in the main figures are available in Supplementary Data 1–3.
