# Supplementary material for: Beclin 1 regulates recycling endosome and is required for skin development in mice
Source: Commun Biol. 2019 Jan 25;2:37. doi: 10.1038/s42003-018-0279-0 (PMC6347619; doi:10.1038/s42003-018-0279-0)
Supplement: Supplementary file 1 — Description of Additional Supplementary Files [file 42003_2018_279_MOESM1_ESM.docx]

**Description of Additional Supplementary Files**

**File Name**: Supplementary Data 1

**Description**: Data sources for Fig. 1c and 1d.

**File Name**: Supplementary Data 2

**Description**: Data sources for Fig. 3b and 3c.

**File Name**: Supplementary Data 3

**Description**: Data sources for Fig. 5c
