# Supplementary material for: Beclin 1 regulates recycling endosome and is required for skin development in mice
Source: Commun Biol. 2019 Jan 25;2:37. doi: 10.1038/s42003-018-0279-0 (PMC6347619; doi:10.1038/s42003-018-0279-0)
Supplement: Supplementary file 2 — Supplementary Information [file 42003_2018_279_MOESM2_ESM.pdf]

Supplementary Figure 1 Noguchi et al.

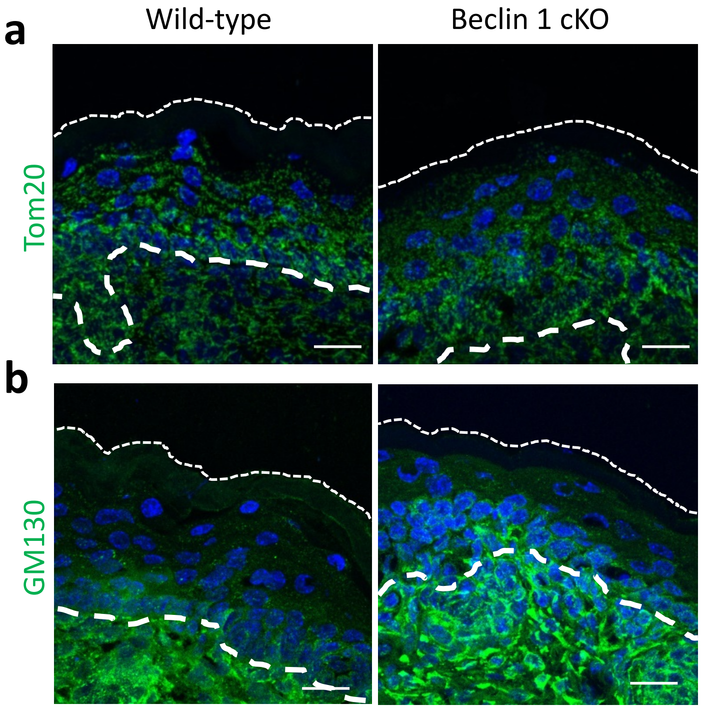

Supplementary Figu re 1. Normal organellar clearance in the epidermis of Beclin 1 cKO mice
Frozen sections (E18.5 embryos) were immunostained with anti-Tom20 **(a)** and anti-GM130 **(b)** antibodies. Nuclei were counterstained with DAPI (blue). Small and large white dotted lines are the cornified layer and basal membrane, respectively. Scale bars = 20 µm. Reduction of mitochondria **(a)** and Golgi apparatus **(b)** were observed even in the epidermis of Beclin 1 cKO mice.

Supplementary Figure 2 Noguchi et al.

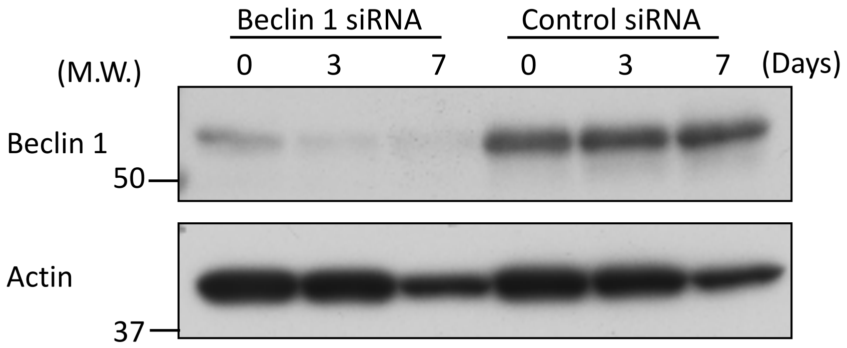

Supplementary Figure 2. Successful downregulation of Beclin 1 in primary keratinocytes
Primary keratinocytes were transfected with the indicated siRNAs. After 3 days, cells were differentiated by the addition of 2 mM $CaCl_2$. Then, cells were harvested and lysed on the indicated days. Immunoblot analysis was performed using the indicated antibodies. Uncropped immunoblot images are provided in Supplementary Figure 5d.

Supplementary Figure 3 Noguchi et al.

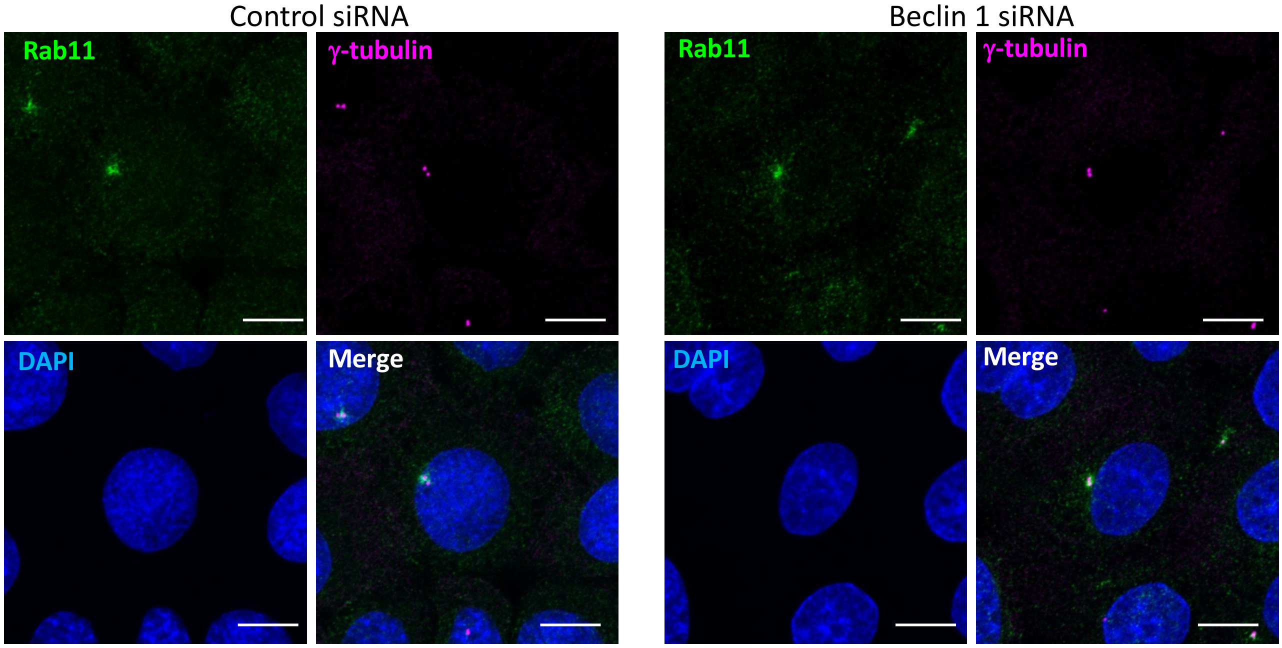

Supplementary Figure 3. Close localization of recycling endosomes to the MTOC
Primary keratinocytes were transfected with the indicated siRNAs. After 3 days, cells were differentiated by the addition of 2 mM $CaCl_2$ for 6 hours. Then, cells were fixed and immunostained with anti-Rab11 (green) and anti-g-tubulin (magenta) antibodies. Nuclei were counterstained with DAPI (blue). Scale bars = 10 µm.

Supplementary Figure 4 Noguchi et al.

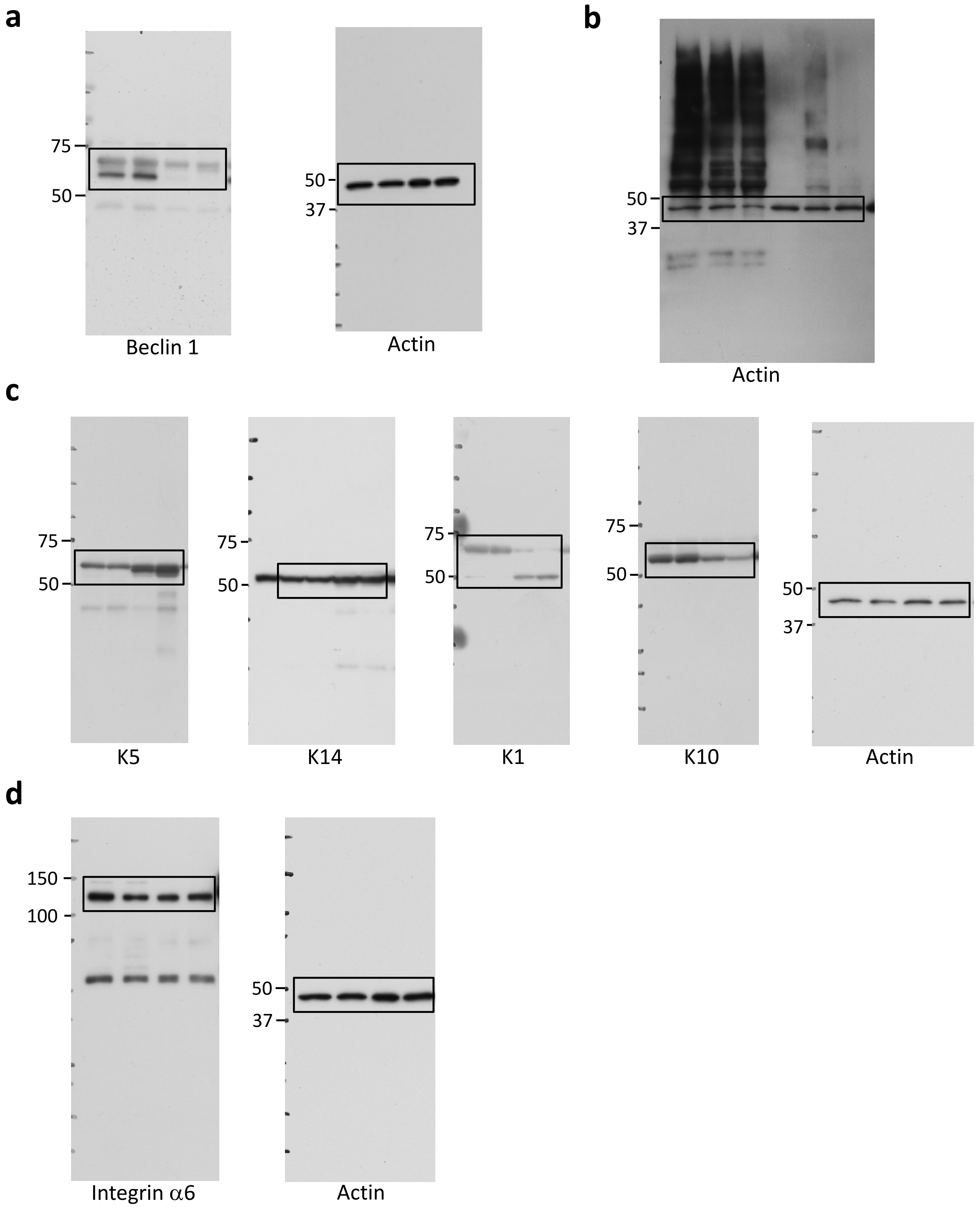

Supplementary Figure 4. Uncropped immunoblot images corresponding to Fig. 1, 2 and 4.

Supplementary Figure 5 Noguchi et al.

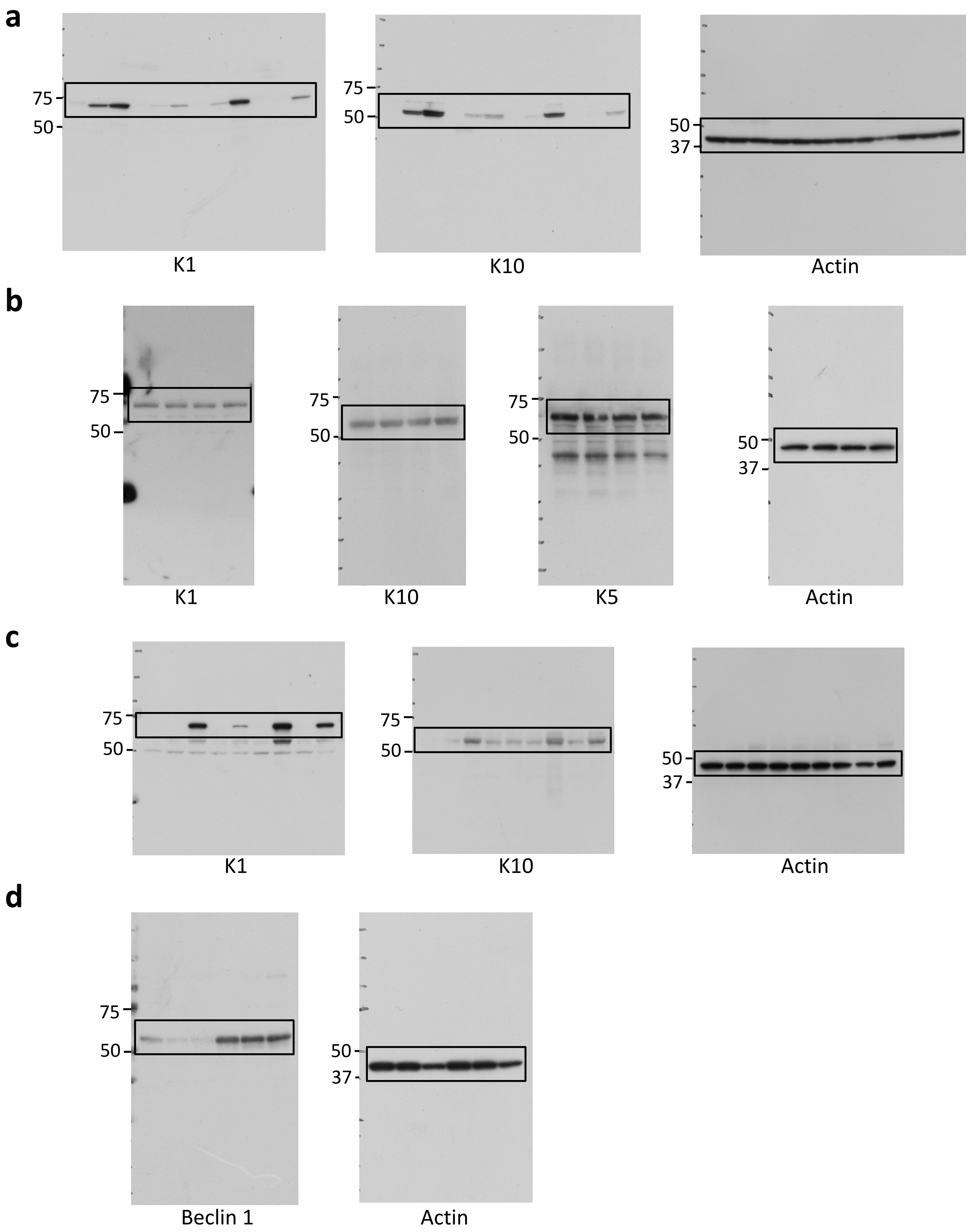

Supplementary Figure 5. Uncropped immunoblot images corresponding to Fig. 5, 6, 7 and Suppl Fig. 2.
